# Identification of Amino Acids in Trm734 Required for 2′-*O*-Methylation of the tRNA^Phe^ Wobble Residue

**DOI:** 10.1021/acsomega.4c02313

**Published:** 2024-06-03

**Authors:** Holly M. Funk, Jennifer H. Brooks, Alisha E. Detmer, Natalie N. Creech, Michael P. Guy

**Affiliations:** Department of Chemistry & Biochemistry, Dorothy Westerman Herrmann Science Center (SC), Room 204F, Northern Kentucky University, Highland Heights, Kentucky 41076, United States of America

## Abstract

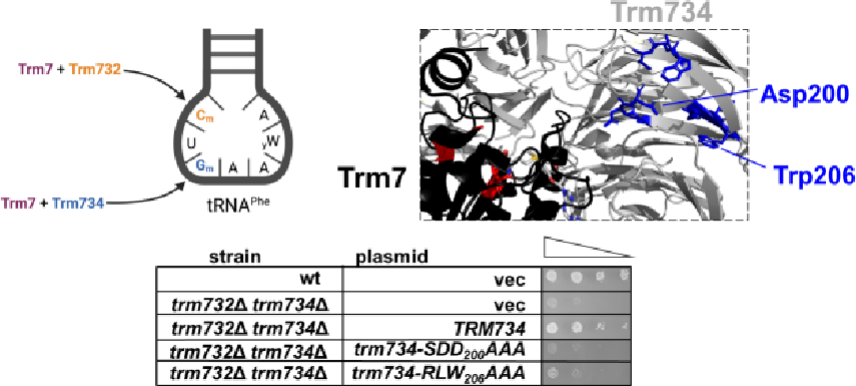

All organisms methylate their nucleic acids, and this methylation is critical for proper gene expression at both the transcriptional and translational levels. For proper translation in eukaryotes, 2′-*O*-methylation of C_32_ (Cm_32_) and G_34_ (Gm_34_) in the anticodon loop of tRNA^Phe^ is critical, with defects in these modifications associated with human disease. In yeast, Cm_32_ is formed by an enzyme that consists of the methyltransferase Trm7 in complex with the auxiliary protein Trm732, and Gm_34_ is formed by an enzyme that consists of Trm7 in complex with Trm734. The role of Trm732 and Trm734 in tRNA modification is not fully understood, although previous studies have suggested that Trm734 is important for tRNA binding. In this report, we generated Trm734 variants and tested their ability to work with Trm7 to modify tRNA^Phe^. Using this approach, we identified several regions of amino acids that are important for Trm734 activity and/or stability. Based on the previously determined Trm7–Trm734 crystal structure, these crucial amino acids are near the active site of Trm7 and are not directly involved in Trm7–Trm734 protein–protein interactions. Immunoprecipitation experiments with these Trm734 variants and Trm7 confirm that these residues are not involved in Trm7–Trm734 binding. Further experiments should help determine if these residues are important for tRNA binding or have another role in the modification of the tRNA. Furthermore, our discovery of a nonfunctional, stable Trm734 variant will be useful in determining if the reported roles of Trm734 in other biological processes such as retromer processing and resistance to Ty1 transposition are due to tRNA modification defects or to other bona fide cellular roles of Trm734.

## Introduction

Nucleic acids are methylated in all domains of life,^1^ and these modifications are critical for the regulation of gene expression.^2−5^ RNA methylation is particularly varied and widespread,^1,6^ with methylations commonly occurring on rRNA (rRNA),^7^ tRNA (tRNA),^8−10^ and mRNA (mRNA).^11^ Over 150 different types of modified ribonucleosides have been identified, with over 100 of these modified nucleosides containing a methyl modification (often in combination with other modifications).^1^ The greatest diversity and extent of these modifications occur on tRNA.^1,12^

One of the most common and widespread RNA methylations occurs on the 2′ hydroxyl of the ribose moiety of the nucleotide (2′-*O*-methylation).^1^ 2′-*O*-Methylated residues are commonly found in rRNA and tRNA, and these modified nucleotides are important for proper function of these noncoding RNAs,^13^ with defects often causing or linked to human disease.^14^ More recently, 2′-*O*-methylation has been shown to be widespread on mRNA,^15^ with some internal 2′-*O*-methylations playing a role in gene regulation.^16,17^

**Figure 1:**
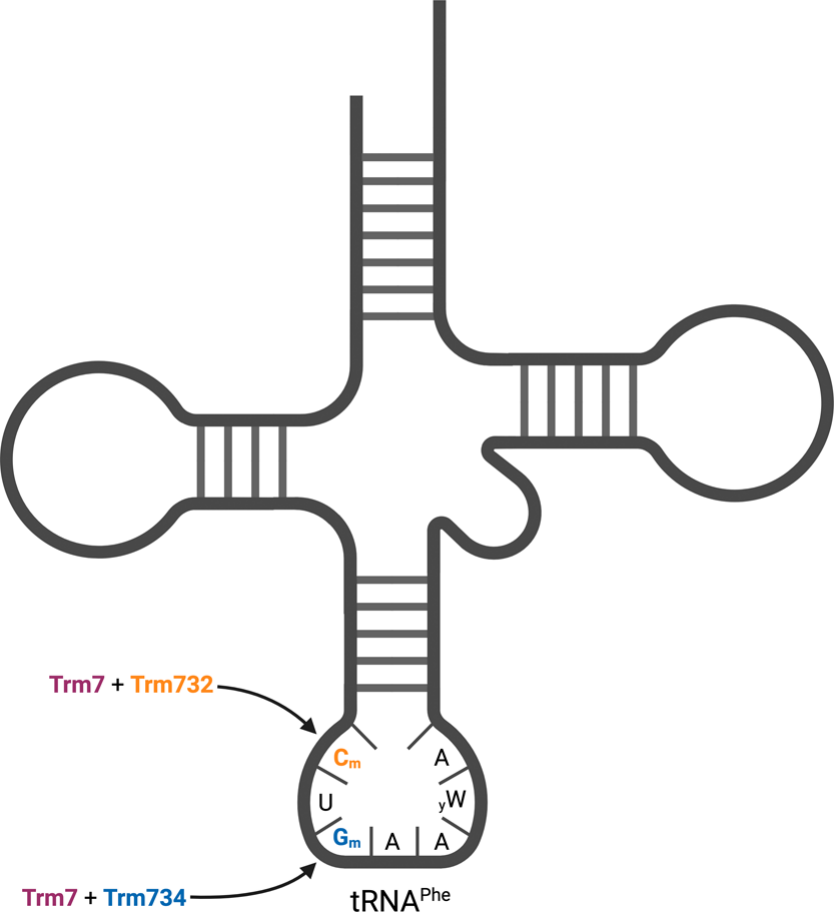
Schematic of 2′-*O*-methylation of the anticodon loop of tRNA^Phe^ in yeast. In yeast, the Trm7–Trm732 complex forms Cm_32_ on tRNA^Phe^, and the Trm7–Trm734 complex forms Gm_34_.

In eukaryotic rRNA and tRNA, formation of 2′-*O*-methylation occurs on specific residues via one of two general mechanisms.^18^ In the first mechanism, 2′-*O*-methylations are formed by small nucleolar RNA protein complexes (snoRNPs), which include the methyltransferase NOP1/fibrillarin, and a box C/D RNA complementary to the target RNA.^19^ This mechanism of 2′-*O*-methylation is commonly observed for rRNA (rRNA)^20,21^ but also occurs on archaeal tRNA^22,23^ and was recently found to occur on eukaryotic tRNA.^24,25^ In the second mechanism, a specific stand-alone methyltransferase modifies a specific nucleic acid residue of target RNA(s).^13^ For example, in the yeast *Saccharomyces cerevisiae*, all five 2′-*O*-methylations found on tRNA are formed by specific methyltransferases rather than by snoRNPs.^26^ Three of these 2′-*O*-methylations are added by the single subunit enzymes Trm3 (Gm_18_ on 10 different substrate tRNAs),^27^ Trm13 (Nm_4_ on three different substrate tRNAs),^28^ and Trm44 (Um_44_ on all four different species of tRNA^Ser^).^29^ The other two 2′-*O*-methylations are added to substrate tRNAs by Trm7,^30^ which binds with Trm732 to form the active methyltransferase responsible for 2′-*O*-methylation of residue 32 (Nm_32_) and separately with Trm734 to form the active methyltransferase responsible for 2′-*O*-methylation of residue 34 (Nm_34_)^31^ (Figure 1). These modifications occur on tRNA^Phe^, tRNA^Trp^, and tRNA^Leu(UAA)^ in *S. cerevisiae*.^30^ The presence or absence of Trm732 has no obvious effect on Nm_34_ levels, and the presence or absence of Trm734 has no obvious effect on Nm_32_ levels on these substrate tRNAs.^31^

Formation of Nm_32_ and Nm_34_ on substrate tRNAs by Trm7 and its partner proteins is conserved in the eukaryotes that have been analyzed and appears to be important for proper translation.^30−38^ In the yeasts *S. cerevisiae* and *Schizosaccharomyces pombe*, lack of these modifications on tRNA^Phe^ causes a slow growth defect and robust activation of the general amino acid control (GAAC) response.^31,34,39^ The Trm7 homologue in humans, mice, and fruit flies is FTSJ1,^32,34,35,37^ and mutations and deletions of the gene encoding this methyltransferase cause nonsyndromic X-linked intellectual disability in humans^33,35^ and pleiotropic effects in flies and mice.^32,36,38^ Deletion of only *TRM732* or only *TRM734* in *S. cerevisiae* and subsequent loss of only Nm_32_ or only Nm_34_ do not cause an obvious phenotypic defect.^31,34,39^ In *S. pombe*, deletion of *TRM732* does not result in an observable deleterious phenotype, but deletion of *TRM734* and subsequent loss of Gm_34_ on tRNA^Phe^ cause a growth defect and activation of the GAAC response.^34,39^

The requirement of specific auxiliary proteins for methylation activity appears to be unique for Trm7 among the FTSJ family of proteins. Members of the FTSJ family are responsible for specific 2′-*O*-methylation of residues in target stem-loop structures of rRNA or tRNA.^40^ All FTSJ family members except for Trm7 methylate either the first or second residue of a 5-base loop of stem-loop structures of the large subunit of bacterial, mitochondrial, and cytosolic rRNA, apparently without a designated partner protein.^41−44^ In contrast, Trm7 methylates the first (residue 32) and third (residue 34) residues in a 7-base loop of the tRNA anticodon stem-loop.^30^ Thus, the requirement for Trm732 and Trm734 for Trm7 activity may be related to the difference in number and type of substrates for Trm7.^40^

In addition to the roles of Trm732 and Trm734 in eukaryotic tRNA methylation, they also appear to have other biological roles. THADA, the human Trm732 homologue,^34^ plays a role in thermogenesis in fruit flies^45^ and cold resistance in plants.^46^ In *S. cerevisiae*, *TRM734* was first identified in a screen for genes important for protecting the genome from Ty1 integration and was therefore given the name *RTT10.*(47) Additionally, *TRM734* was also found in a screen for canavanine resistance and has been reported to play a role in endosomal recycling in *S. cerevisiae* by binding to the protein Ere1.^48^ The human Trm734 homologue WDR6 has been reported to be part of an E3 ubiquitin ligase complex in humans^49,50^ and was recently reported to bind to the catalytic subunit of serine/threonine-protein phosphatase 1 (PPP1CB), thus driving lipogenesis in the liver of insulin-resistant mice.^51^ The role of tRNA modification activity in any of these additional biological roles for Trm734 or its homologues has not been investigated.

The exact roles of Trm732 and Trm734 for 2′-*O*-methylation are not known. We recently showed that motif 2 of Trm732, which consists of the highly conserved residues RRSAGLP_754_, is required for Nm_32_ activity by the Trm7–Trm732 complex.^52^ The crystal structure of the Trm7–Trm732 complex has not yet been solved, but the crystal structure of the Trm7–Trm734 complex was solved at a resolution of 2.32 Å.^53^ In the structure, Trm734 was found to be a WD repeat protein with three separate domains that were named BPA, BPB, and BPC, with BPA and BPC directly interacting with Trm7, which is a Rossman fold methyltransferase.^53^ Modeling of tRNA in the structure suggests that Trm734 helps to correctly position the tRNA for modification.^53^ A role of Trm734 in tRNA binding is further supported by the finding that human WDR6 by itself or in complex with human FTSJ1 binds tRNA, whereas FTSJ1 by itself does not.^37^ However, the precise residues and/or motifs of Trm734 required for 2′-*O*-methylation activity are not known.

In this report, we used sequence alignments of Trm734 proteins to identify regions of Trm734 required for modification activity. By generating and testing Trm734 variants, we identify three important, conserved stretches of amino acids in Trm734 that are required for Gm_34_ modification on tRNA^Phe^. We further show that two of these Trm734 variants are relatively stable and can still bind to Trm7. Consistent with these findings, mapping of these residues on the crystal structure shows that they are located in proximity to the active site of Trm7 but do not interact with Trm7 itself. Further experiments with Trm7–Trm734 protein complexes and tRNA should shed light on the role of these residues in tRNA modification.

## Results

### Conserved Residues in Trm734 Are Required for Gm_34_ Modification on tRNA^Phe^

**Figure 2:**
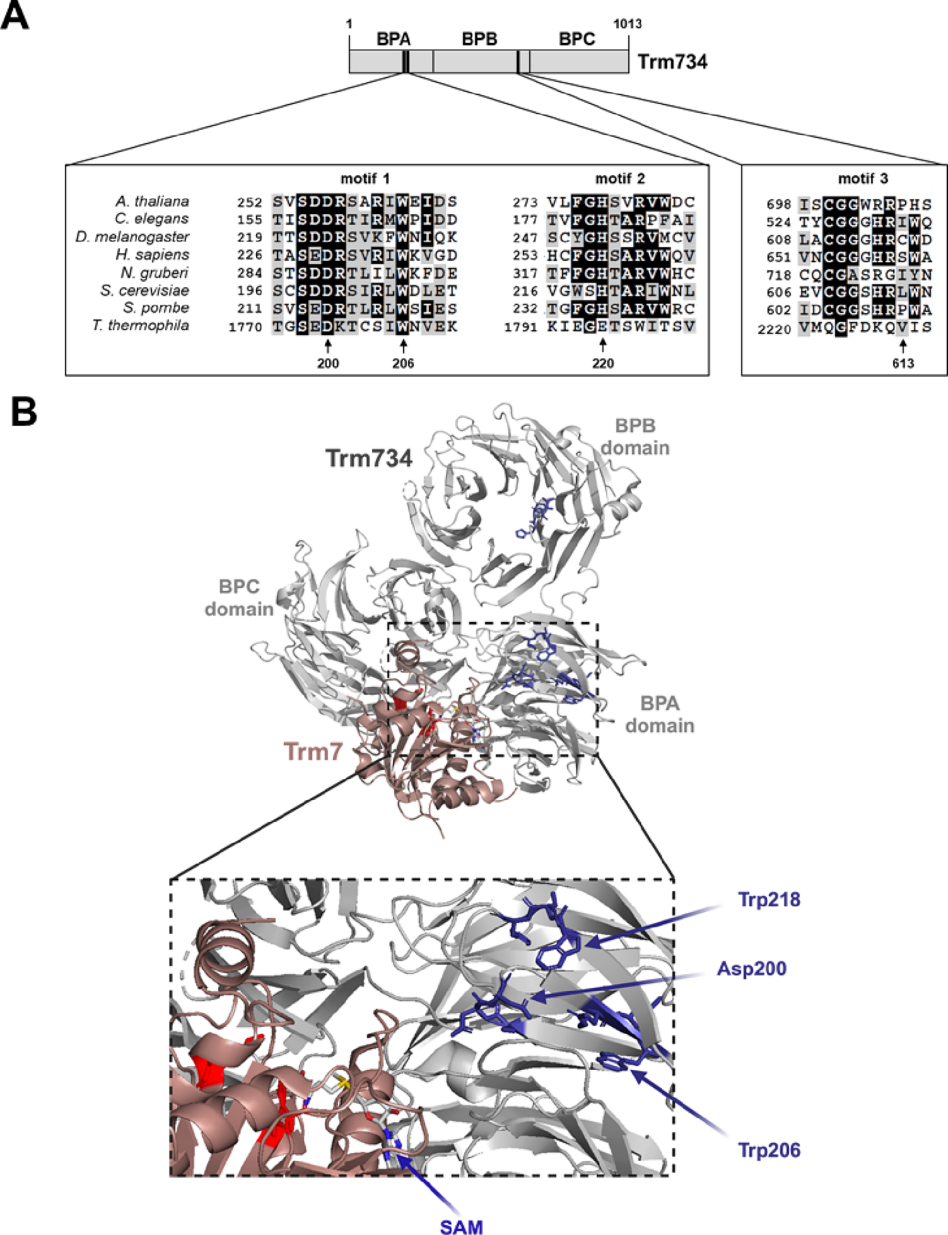
Conserved motifs of Trm734 near the Trm7–Trm734 interface. (A) Schematic representation of Trm734 sequence. Inset box is an amino acid alignment of regions of high sequence similarity between Trm734 proteins from eight diverse eukaryotes. Arrows designate amino acids changed in Trm734 variants analyzed in this study. (B) Location of conserved amino acids in Trm734. Representation of the *S. cerevisiae* Trm7–Trm734 crystal structure (PDB 6JPL). Trm7 is represented as the salmon color with catalytic residues in red. Trm734 is gray, with conserved residues studied represented in blue. *S*-Adenosyl methionine (SAM) is colored by element.

**Figure 3:**
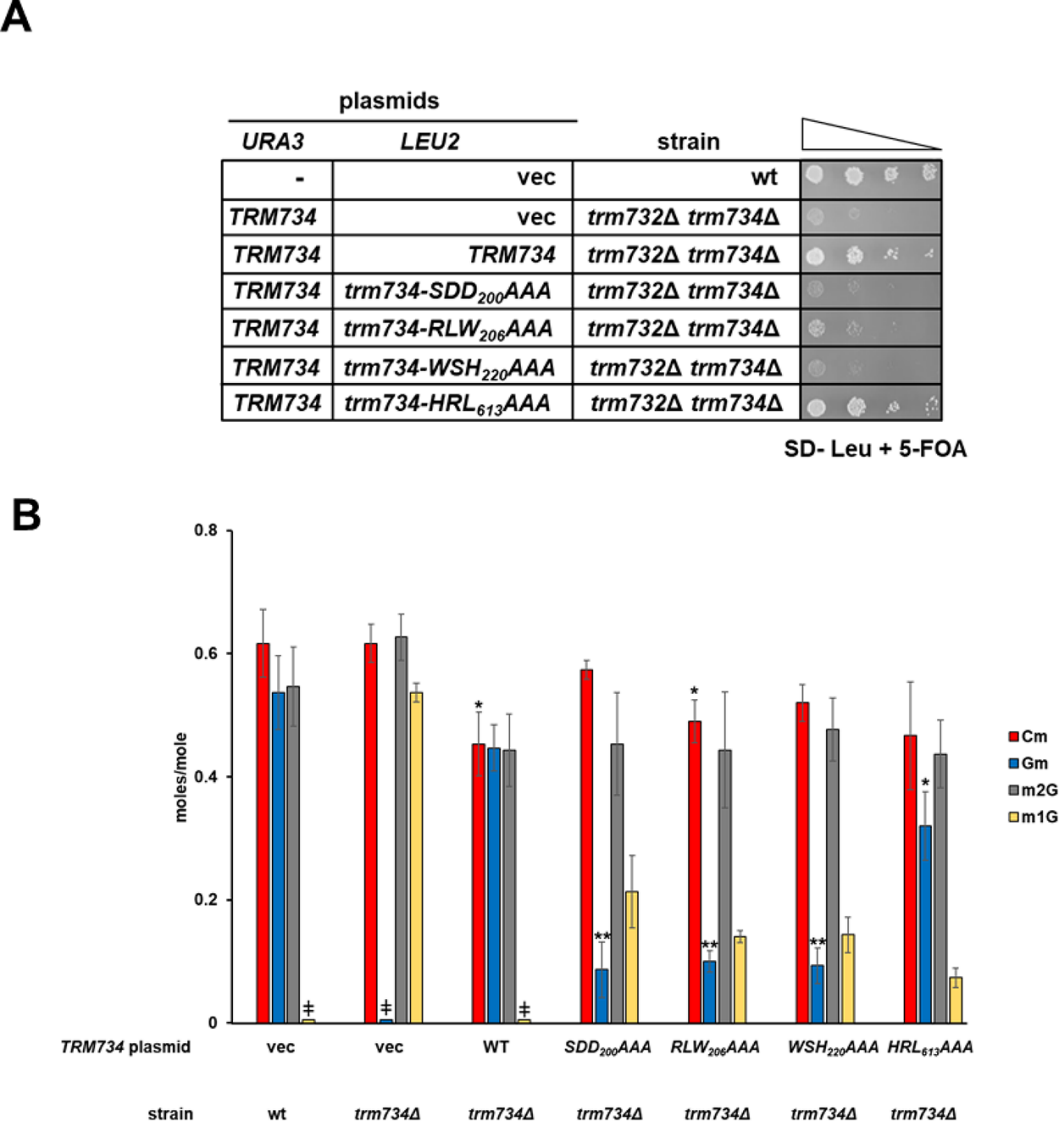
Identification of Trm734 variants defective for Gm_34_ formation on tRNA^Phe^. (A) Several conserved amino acids in Trm734 are required for suppression of the slow growth of *trm732Δtrm734*Δ mutants. Indicated strains containing *URA3* and *LEU2* plasmids were grown overnight in SD- Leu medium, diluted to an OD600 of ∼0.5, serially diluted 10-fold, and then spotted on a medium containing 5-FOA to select against the *URA3* plasmid. Cells were grown for 2 days at 30 °C. (B) Conserved amino acids in Trm734 are required for Gm_34_ formation on tRNA^Phe^ in yeast. Quantification of nucleosides by UPLC from tRNA^Phe^ purified from indicated yeast strains. Bars represent mean and standard deviation from three individual growths and RNA preparations. Statistical significance for each modification compared to wild-type cells expressing a vector control is indicated by asterisks (*P < 0.05, **P < 0.001). m1G in wild-type cells is below the threshold of detection. The symbol ‡ indicates levels below the threshold of detection.

**Figure 4:**
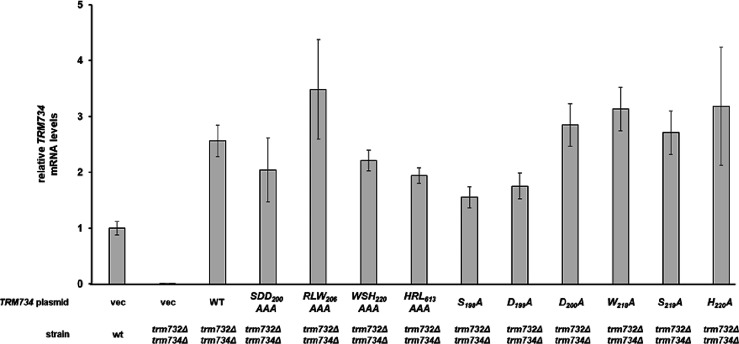
Relative mRNA levels of mutant *TRM734* genes. Graphical representation of Table 2.

To further understand the role of Trm734 in the formation of Nm_34_ by Trm7 on tRNA, we identified conserved amino acid residues, generated Trm734 variants of these residues, and tested the variants for Gm_34_ formation activity. First, to identify candidate amino acid residues, we aligned predicted Trm734 homologues from eight diverse eukaryotes (Figure 2A). We also mapped these residues onto the Trm7–Trm734 crystal structure (Figure 2B).^53^ We found several stretches of conserved amino acids in the 197–210 region of the BPA domain of Trm734, near the Trm7 active site (Figure 2A,B). We generated Trm734 variants that replaced these conserved residues with alanine residues in a low-copy *CEN* plasmid for expression yeast. To test the result of changing conserved amino acid residues on Trm734 activity, we first performed a genetic test of function. Because the presence of either Gm_34_ or Cm_32_ alone on tRNA^Phe^ is sufficient for healthy growth in *S. cerevisiae*,^31^ we could not use a *trm734*Δ single mutant strain to test the function of Trm734 variants by observing complementation of a growth defect. We therefore tested the ability of Trm734 variants to rescue the slow growth of the *trm732*Δ*trm734*Δ double mutant strain. We expressed the Trm734 variants in the *trm732Δtrm734*Δ [*TRM734 URA3*] strain and analyzed growth after plating on media containing 5-FOA to select against the [*TRM734 URA3*] plasmid. *trm732Δtrm734*Δ strains expressing wild-type Trm734 or Trm734-HRL_613_AAA were healthy (Figure 3A). In contrast, strains expressing the Trm734-SDD_200_AAA and Trm734-WSH_220_AAA variants grew similarly to strains not expressing Trm734, indicating that those residues were important for Trm734 function (Figure 3A). A strain expressing the Trm734-RLW_206_AAA variant showed only a slight increase in growth compared to cells expressing a vector (Figure 3A), indicating that these amino acids are also important for Trm734 function. There is no commercially available antibody for *S. cerevisiae* Trm734; therefore, we could not perform Western blot analysis to determine protein levels for each variant. However, to verify that *TRM734* mRNA was produced for each of the variants, we performed quantitative real-time PCR (qRT-PCR). mRNA from all *TRM734* gene variants tested was expressed at levels comparable to or greater than chromosomal *TRM734* from wild-type cells (Table 1, Figure 4), indicating that the loss of complementation by variants was not due to the loss of gene transcription.

**Table 1:** Relative mRNA Levels of Mutant *TRM734* Genes

**strain**	**plasmid**	**relative levels**^a^
wild type	vec	1.00 ± 0.12
*trm732Δtrm734*Δ	vec	0.001 ± 0.0006
*trm732Δtrm734*Δ	*TRM734*	2.56 ± 0.28
*trm732Δtrm734*Δ	*TRM734-SDD*_*200*_*AAA*	2.04 ± 0.57
*trm732Δtrm734*Δ	*TRM734-RLW*_*206*_*AAA*	3.48 ± 0.89
*trm732Δtrm734*Δ	*TRM734-WSH*_*220*_*AAA*	2.21 ± 0.18
*trm732Δtrm734*Δ	*TRM734-HRL*_*613*_*AAA*	1.94 ± 0.14
*trm732Δtrm734*Δ	*TRM734-S*_*198*_*A*	1.55 ± 0.19
*trm732Δtrm734*Δ	*TRM734-D*_*199*_*A*	1.75 ± 0.23
*trm732Δtrm734*Δ	*TRM734-D*_*200*_*A*	2.85 ± 0.38
*trm732Δtrm734*Δ	*TRM734-W*_*218*_*A*	3.13 ± 0.39
*trm732Δtrm734*Δ	*TRM734-S*_*219*_*A*	2.71 ± 0.39
*trm732Δtrm734*Δ	*TRM734-H*_*220*_*A*	3.18 ± 1.06

a Relative to *TRM734* in wild-type cells after normalization to *ACT1*. Values are from three independent growths.

We next measured the levels of Gm on tRNA^Phe^ from *trm734*Δ strains expressing the Trm734 variants using UPLC. We chose to test the Trm734 variants in *trm734*Δ single mutants because a growth defect is not required to biochemically analyze the tRNA modification levels in mutants expressing the variants. We found that the ability of Trm734 variants to rescue the slow growth of the *trm732Δtrm734*Δ double mutant generally corresponded to levels of Gm_34_ on tRNA^Phe^ from the *trm734*Δ mutants. Thus, *trm734*Δ strains expressing the Trm734-HRL_613_AAA variant had lower amounts of Gm_34_ on tRNA^Phe^ compared to that in wild-type yeast (0.32 vs 0.54 mol/mol) (Figure 3B). Although this is a statistically significant decrease, mutant strains expressing Trm734-SDD_200_AAA (0.09 mol/mol), Trm734-RLW_206_AAA (0.1 mol/mol), and Trm734-WSH_220_AAA (0.09 mol/mol) had much lower levels of Gm on their tRNA^Phe^ compared to that of wild type. Because variants were expressed in a strain expressing Trm732, levels of Cm were similar in all strains regardless of the presence or absence of a functional Trm734 protein (Figure 3B). A slight, statistically significant decrease in Cm_32_ on tRNA^Phe^ from mutants expressing wild-type Trm734 (0.45) or the Trm734-RLW_206_AAA variant (0.49) was observed compared to that from wild-type cells (0.62 mol/mol). Significant levels of m^1^G were detected in strains with defective levels of Gm but not in other strains. Detection of m^1^G in these strains is expected because m^1^G_37_ is a precursor to yW_37_ on tRNA^Phe^, and *trm734*Δ mutants have been shown previously to have yW defects.^31^ Therefore, decreased levels of Gm_34_ in mutant strains lead to reduced levels of yW, resulting in the increased levels of m^1^G. Levels of m^2^G on tRNA^Phe^ were relatively constant in each strain, as expected for a modification that does not require Trm734 for formation and is not affected by Gm_34_ levels. These results further indicate that residues SDD_200_, RLW_206_, and WSH_220_ are critical for Trm734 function and are consistent with the results obtained in the growth assays for the variants.

**Figure 5:**
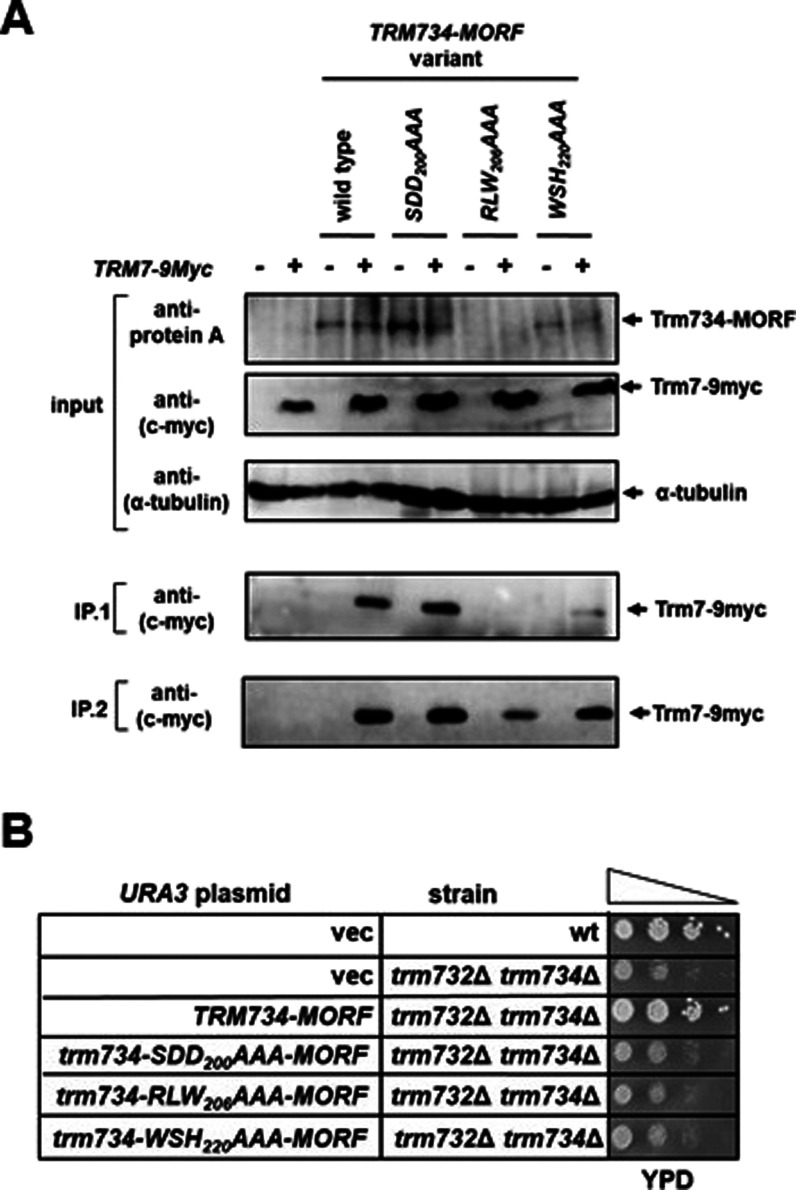
Binding of Trm734 variants to Trm7. (A) Amino acids SDD in motif 1 are not required for Trm7–Trm734 interaction. Protein extracts from indicated strains were grown in SD-Leu medium, and MORF-tagged proteins were purified with IgG-Sepharose beads (which bind the ZZ domain of the MORF tag) as described in Methods. Samples were then analyzed by SDS-PAGE and immunoblot. IP.1 and IP.2 correspond to two independent immunoprecipitation trials. (B) MORF-tagged Trm734 variants behave similarly to untagged variants in growth assays. Indicated strains containing *URA3* plasmids were grown overnight in SD-Ura medium, diluted to an OD600 of ∼0.5, serially diluted 10-fold, and then spotted on rich media. Cells were grown for 2 days at 30 °C.

The lowered levels of Gm_34_ formation on tRNA^Phe^ observed for the Trm734-SDD_200_AAA, Trm734-RLW_206_AAA, and Trm734-WSH_220_AAA variants could be due to any of several different defects including reduced protein stability, reduced Trm734 binding to Trm7, reduced tRNA binding, reduced Trm7–Trm734 catalytic activity due to problems in positioning of G_34_, or other defects. To determine if the loss of activity in these variants was due to the loss of protein stability, we expressed MORF-tagged Trm734 variants from a low-copy CEN plasmid and performed immunoblot analysis. Immunoblot analysis indicates that the Trm734-SDD_200_AAA variant was expressed at least to the same level as wild-type Trm734, whereas the Trm734-WSH_220_AAA variant seemed to have a significantly lowered expression (Figure 5A). We failed to detect the expression of the Trm734-RLW_206_AAA variant using this approach (Figure 5A). Thus, it is likely that at least part of the defect observed in the Trm734-RLW_206_AAA, and Trm734-WSH_220_AAA variants is due to the loss of Trm734 stability. Because it is possible that the MORF tag used in the pulldown could have stabilized the Trm734 variants, we also determined whether MORF-tagged Trm734 variants were able to rescue the slow growth of the *trm732Δtrm734*Δ variant. We found that MORF-tagged Trm734-SDD_200_AAA, Trm734-RLW_206_AAA, and Trm734-WSH_220_AAA variants grew similarly to strains not expressing Trm734, whereas MORF-tagged wild-type Trm734 complemented the growth defect (Figure 5B), similar to what we observed for the untagged proteins. These results indicate that the addition of the MORF tag did not significantly stabilize the variants.

We next performed an immunoprecipitation assay to determine whether the Trm734 variants could bind to Trm7. To this end, we coexpressed MORF-tagged Trm734 variants with 9myc-tagged Trm7. We found that the tagged Trm734-SDD_200_AAA variant bound Trm7-9myc at similar levels to wild-type Trm734 (Figure 5A) regardless of the experiment and exposure time of the blot, strongly suggesting that the lack of Gm_34_ formation activity of this variant is not due to lowered levels of protein or loss of Trm7 binding. In an immunoprecipitation experiment and subsequent blot with low exposure time, less Trm7-9myc was detected in pulldown by Trm734-WSH_220_AAA as compared to the wild type. This lower amount of pulldown could be due to the lower levels of the Trm734 variant observed (Figure 5A). Trm7–9myc pulldown by the Trm734-W_206_AAA variant was significantly lower than that of wild type, which is consistent with the difficulty in detecting the expression of this Trm734 variant (Figure 5A). We performed this experiment multiple times, with results for two of these replicate experiments shown (Figure 5A).

**Figure 6:**
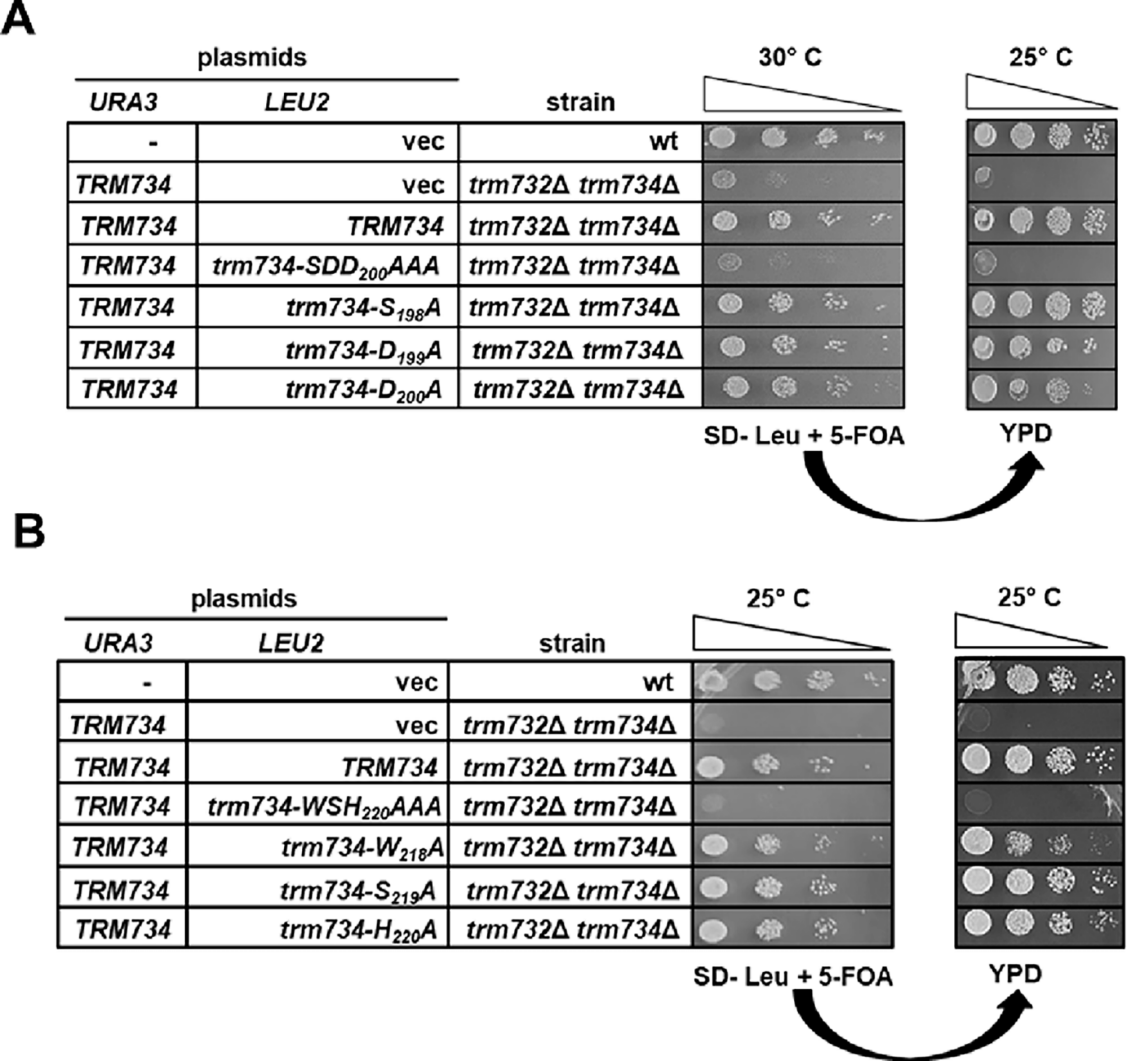
Requirement of individual motif 1 and 2 residues for Trm734 function. (A) Amino acid residue D200 in motif 1 is important for Trm734 function. Strains with plasmids as indicated were grown overnight in SD-Leu and analyzed as in Figure 3A after incubation for 2 days at 30 °C. Following growth on 5-FOA at 30 °C, cells were spotted on YPD at 25 °C and incubated for 2 days. (B) Amino acid residue W218 in motif 2 is important for Trm734 function. Strains with plasmids as indicated were grown overnight in SD-Leu and analyzed as in Figure 3A after incubation for 2 days at 25 °C. Following growth on 5-FOA, cells were spotted on YPD at 25 °C and incubated for 2 days.

To further analyze the roles of selected individual amino acids in the activity of Trm734, we made variants with single amino acid substitutions in the Trm734 SDD_200_ and Trm734 WSH_220_ motifs and tested their ability to rescue the slow growth of the *trm732Δtrm734*Δ double mutant strain. We found that the expression of the Trm734-S_198_A and Trm734-D_199_A single amino acid variants was fully able to suppress the growth defect of the *trm732Δtrm734*Δ strain, whereas Trm734-D_200_A showed a slight growth defect as determined by a spot test (Figure 6A) and verified by a liquid growth assay (Table 2). This growth defect is likely due to lowered Gm_34_ levels on tRNA^Phe^, suggesting that residue D_200_ is important for the Gm_34_ activity of the Trm7–Trm734 enzyme or the stability of the Trm734 protein. Likewise, expression of the Trm734-S_219_A and Trm734-H_220_A single amino acid variants fully suppressed the growth defect of the *trm732Δtrm734*Δ strain, whereas Trm734-W_218_A showed a slight growth defect at room temperature by a spot test (Figure 6B) or by a liquid growth assay (Table 2).

**Table 2:** Comparison of Generation Times for *trm732Δ trm734*Δ Mutants Expressing Trm734 Variants in Minimal Media at 26°C

**strain**	*TRM734***plasmid**	**generation time (min)**^a^
wild type	vec	191 ± 3
*trm732Δtrm734*Δ	vec	556 ± 70
*trm732Δtrm734*Δ	*TRM734*	195 ± 2
*trm732Δtrm734*Δ	*TRM734-SDD*_*200*_*AAA*	575 ± 97
*trm732Δtrm734*Δ	*TRM734-RLW*_*206*_*AAA*	305 ± 12
*trm732Δtrm734*Δ	*TRM734-WSH*_*220*_*AAA*	557 ± 23
*trm732Δtrm734*Δ	*TRM734-S*_*198*_*A*	184 ± 2
*trm732Δtrm734*Δ	*TRM734-D*_*199*_*A*	187 ± 4
*trm732Δtrm734*Δ	*TRM734-D*_*200*_*A*	303 ± 40
*trm732Δtrm734*Δ	*TRM734-W*_*218*_*A*	251 ± 9
*trm732Δtrm734*Δ	*TRM734-S*_*219*_*A*	195 ± 2
*trm732Δtrm734*Δ	*TRM734-H*_*220*_*A*	191 ± 3

a Mean and standard deviations based on growths from three different colonies.

## Discussion

In this study, we have identified amino acid residues in Trm734 that are required for Trm7-dependent formation of Gm_34_ on tRNA^Phe^. These important residues are found in a location on the Trm7–Trm734 complex that could feasibly interact with a bound tRNA molecule based on modeling.^53^ The data thus suggest that a role of these amino acids could be to bind the tRNA and possibly position it properly for specific modification of residue 34 by Trm7. Alanine substitution for residues SDD_200_ did not perturb Trm734 protein stability or its ability to bind to Trm7, suggesting an important role of these residues for tRNA binding or for another function.

Our genetic and biochemical data demonstrate that Trm734 residues, SDD_200_ and WSH_220_, are required for tRNA modification by the Trm7–Trm734 complex (Figure 3). Based on the crystal structure of the Trm7–Trm734 complex, it is clear that these residues are not involved in the interaction between Trm7 and Trm734 (Figure 2),^53^ which was further confirmed by our immunoprecipitation results (Figure 5). Alanine substitution of RLW_206_, and, to a lesser extent, WSH_220_ appeared to affect protein stability, at least when these proteins carried a MORF tag. Although the structure of the Trm7–Trm734 complex bound to tRNA has not been determined, the location of these residues suggests that they could be involved in binding and/or correctly positioning the tRNA in the active site of Trm7. The SDD and WSH residues are found near each other in a region of β-turns relatively close to the active site of Trm7 (Figure 2),^53^ further supporting this idea. The exact role of the negatively charged SDD residues in modification is not clear, although they are important based on our data and their high conservation among eukaryotic Trm734 proteins (Figure 2).

Our finding that Trm734 SDD_200_AAA is a stable variant capable of binding to Trm7 will be a useful tool in determining whether Trm734 has functions in yeast and other eukaryotes beyond being involved in the formation of Nm_34_ on substrate tRNAs. *TRM734* is also known by the aliases *RTT10* and *ERE2* due to its initial discovery in a screen for defense against Ty1 transposition^47^ and a role in endosomal recycling,^48^ respectively. Rescue of defects in endosomal recycling by expression of the Trm734 SDD_200_AAA variant in *trm734*Δ mutants would show that Trm734 has a role in this process not associated with its tRNA modification activity. Likewise, a similar approach with this variant could be used to determine if Trm734 has a direct role in defense against Ty1 transposition.^47^

Because the Trm734 residues that we found to be important for function are highly conserved, these results will likely be useful to study how human Trm7 (FTSJ1) works with human Trm734 (WDR6) to modify substrate tRNAs and whether these proteins also function directly in other biological processes. Humans lacking *FTJS1* have intellectual disability,^33^ and *FTJS1* KO mice exhibit abnormalities in their synapses and accompanying memory deficits and anxiety-like behavior.^38^ Because FTSJ1 is responsible for both the Nm_32_ and Nm_34_ modifications, it is unclear whether these phenotypes are due to the lack of both modifications or just one. Because FTSJ1 requires WDR6 for modification of tRNA residue 34,^37^ analysis of WDR6 variants should help determine which modifications are most important in these processes. However, phenotypic analyses of *WDR6* mutants in mice and humans are complicated by the finding that WDR6 is purported to be involved in processes including lipogenesis,^51^ protein degradation through E3 ubiquitin ligase binding,^49,50^ and viral defense.^54^ Use of a tRNA-modification deficient WDR6 variant in these experiments could ensure that only effects of loss of tRNA modification activity are tested and could also help determine if any of the other biological roles of WDR6 are related to the tRNA modification activity.

## Methods

### Yeast Strains and Plasmids

Yeast strains are listed in Table 3. The *trm734Δ::ble*^*R*^, *trm732Δ::ble*^*R*^, *trm734Δ::ble*^*R*^*trm732Δ::kanMX* [*CEN URA3 TRM734*], and Trm7–9myc strains were constructed using standard techniques, as described previously.^31^ Plasmids are listed in Table 4. The *CEN LEU2 TRM734* expression plasmid was constructed by PCR amplification of *TRM734* from pBP2A^31^ with primers that introduced *Nde*I and *Bam*HI cut sites followed by ligation into plasmid pAVA581^55^ at those sites. Plasmids expressing Trm734 variants were generated by Q5 site-directed mutagenesis (New England Biolabs). The *CEN URA3 TRM734-MORF* plasmid was generated by amplification of the MORF tag from the 2 μ C-terminal tagged *TRM7-MORF* plasmid^56^ and addition to pBP2A (*CEN URA3 TRM734*)^31^ by Gibson assembly.^57^ All plasmids were confirmed by sequencing prior to use.

**Table 3:** Strains Used in This Study

**strain**	**genotype**	**source**
BY4741	*MAT*a *his3-Δ1 leu2Δ0 met15-Δ0 ura3-Δ0*	Open Biosystems
yMG724-5	BY4741, *trm732Δ::ble*^*R*^	(31)
yMG818-1	BY4741, *trm734Δ::ble*^*R*^*trm732Δ::kanMX* [*CEN URA3 TRM734*]	(31)
yMG842-1	BCY123, *TRM7–9myc::kanMX*	(31)

**Table 4:** Plasmids Used in This Study

**plasmid**	**parent**	**description**	**source**
pBP2A		*CEN URA3 TRM734*	(31)
pAVA581		*CEN LEU2 LIC*	(55)
pMG683B	pBP2A	*CEN LEU2 TRM734*	this study
pMG705B	pMG683B	*CEN LEU2 TRM734-SDD*_*200*_*AAA*	this study
pMG714A	pMG683B	*CEN LEU2 TRM734-RLW*_*206*_*AAA*	this study
pMG715A	pMG683B	*CEN LEU2 TRM734-WSH*_*220*_*AAA*	this study
pMG716C	pMG683B	*CEN LEU2 TRM734-HRL*_*613*_*AAA*	this study
pMG723F	pMG683B	*CEN LEU2 TRM734-S*_*198*_*A*	this study
pMG736A	pMG683B	*CEN LEU2 TRM734-D*_*199*_*A*	this study
pMG724A	pMG683B	*CEN LEU2 TRM734-D*_*200*_*A*	this study
pMG727F	pMG683B	*CEN LEU2 TRM734-W*_*218*_*A*	this study
pMG737M	pMG683B	*CEN LEU2 TRM734-S*_*219*_*A*	this study
pMG738B	pMG683B	*CEN LEU2 TRM734-H*_*220*_*A*	this study
pMG771A	pBP2A	*CEN URA3 TRM734-MORF*	this study
pMG811B	pMG771A	*CEN URA3 TRM734-SDD*_*200*_*AAA-MORF*	this study
pMG812C	pMG771A	*CEN URA3 TRM734-RLW*_*206*_*AAA-MORF*	this study
pMG813B	pMG771A	*CEN URA3 TRM734-WSH*_*220*_*AAA-MORF*	this study

### Isolation of RNA from Yeast Cells

*S. cerevisiaetrm734*Δ strains harboring *CEN* plasmids expressing Trm734 variants were grown in liquid dropout media to an OD of ∼2. RNA was extracted using the hot phenol method.^58^

### Quantitative Real-Time PCR

RNA was first treated with RNase-free DNase (Promega) and then reverse transcribed using a Verso cDNA Kit (Thermo Scientific) with a 3:1 (v/v) mix of random hexamers and anchored oligo-dT primers. cDNA was then amplified using a DyNAmo HS SYBR Green qPCR Kit (Thermo Scientific) with primers specific to indicated genes. Oligonucleotides used to detect *TRM734* mRNA are specific to a region of the gene that was not mutated in any of the variants. Oligonucleotides used are given in Table 5.

**Table 5:** Oligonucleotides Used for This Study

**primer**	**target**	**sequence**
MPG1598	*ACT1*	GAAATGCAAACCGCTGCTCA
MPG1599	*ACT1*	TACCGGCAGATTCCAAACCC
MPG1950	*TRM734*	TTGACCACAAACTGGACGCT
MPG1951	*TRM734*	TGGCGGAAGTTCTTGTAGCA

### Purification of tRNA and Analysis of Modified Nucleosides by UPLC

Yeast tRNA^Phe^ was isolated using a complementary biotinylated oligonucleotide followed by tRNA digestion with P1 nuclease and phosphatase as previously described.^58^ After digestion, nucleosides were analyzed by UPLC using a 50 mm HSS T3 C_18_ column with a 1.8 μM particle size. The buffer system consisted of buffer A (5 mM NaOAc pH 7.1 + 0.1% acetonitrile) and buffer B (60% ACN). At a flow rate of 0.46 mL/min, the gradient was as follows: 98% buffer A for 8.92 min; a gradient to achieve 10% buffer B at 15.45 min; and a gradient to achieve 25% buffer B at 29.73 min followed by 100% buffer B for 2 min. Analyses were performed using triplicate growths and RNA samples.

Nucleosides were quantified by determining the area under the curve for each peak at the maximum absorbance of the nucleoside. The total number of moles of tRNA in each injection was determined by dividing the area under the curve of each standard nucleoside (A, C, G, and U) by its extinction coefficient and normalizing for the expected number of that nucleoside in tRNA^Phe^. Moles of modified nucleoside in the injection were then determined by dividing the area under the curve for each peak by its extinction coefficient and comparing to the moles of tRNA to determine the ratio of modified nucleoside per tRNA, all as described previously.^58^

### Affinity Purification of Tagged Trm7 and Immunoblot Analysis

Trm734-MORF variants were affinity purified with IgG sepharose essentially as previously described^55^ and then eluted by incubation in SDS sample buffer. Yeast crude extracts and affinity purified samples were subjected to SDS-PAGE, and proteins were transferred to a nitrocellulose membrane. Proteins were then detected with the appropriate antibodies. MORF-tagged proteins were detected with rabbit polyclonal antiprotein A (Sigma, 1:5,000) followed by incubation with goat antirabbit IgG-HRP (Bio-Rad, 1:10,000). The 9myc tag was detected with mouse monoclonal anti[c-myc] (Sigma, 1:10,000) followed by incubation with goat antimouse IgG-HRP (Bio-Rad, 1:10,000). α-Tubulin was detected using rabbit monoclonal anti-α-tubulin (Abcam, 1:10,000) followed by incubation with goat antirabbit IgG-HRP (Bio-Rad, 1:10,000).

### Determination of Generation Times for *trm732Δtrm734*Δ Mutants Expressing Trm734 Variants

Wild-type and *trm732Δtrm734*Δ strains with indicated plasmids were grown in triplicate to saturation in selective media and then diluted to an OD of 0.02 in minimal media in a 96-well plate. Growth was monitored by measuring OD every 30 min for 24 h at 26 °C in an Agilent BioTek Synergy H1 Spectrophotometer with continuous orbital shaking.
